# ENDOSCOPIC RETROGRADE CHOLANGIOPANCREATOGRAPHY (ERCP): ANALYSIS OF THE EFFECTIVENESS AND SAFETY OF THE PROCEDURE IN THE PATIENT WITH ROUX-EN-Y GASTRIC BYPASS

**DOI:** 10.1590/0102-672020190001e1432

**Published:** 2019-04-29

**Authors:** Flávio Heuta IVANO, Bruno Jeronimo PONTE, Thais Caroline DUBIK, Victor Kenzo IVANO, Vitória Luiza Locatelli WINKELER, Antônio Katsumi KAY

**Affiliations:** 1 Pontificate Catholic University of Paraná, Medicine; 2 Service of Digestive Endoscopy, Sugisawa Hospital, Curitiba, PR, Brasil

**Keywords:** Endoscopy, Gastric bypass, Cholangiopancreatography, endoscopic retrograde, Endoscopia, Derivação gástrica, Colangiopancreatografia retrógrada endoscópica

## Abstract

**Background:**

Obesity can be treated with bariatric surgery; but, excessive weight loss may lead to diseases of the bile duct such as cholelithiasis and choledocholithiasis. Endoscopic retrograde cholangiopancreatography is a diagnostic and therapeutic procedure for these conditions, and may be hampered by the anatomical changes after surgery.

**Aim:**

Report the efficacy and the safety of videolaparoscopy-assisted endoscopic retrograde cholangiopancreatography technique in patients after bariatric surgery with Roux-en-Y gastric bypass.

**Method:**

Retrospective study performed between 2007 and 2017. Data collected were: age, gender, surgical indication, length of hospital stay, etiological diagnosis, rate of therapeutic success, intra and postoperative complications.

**Results:**

Seven patients had choledocholithiasis confirmed by image exam, mainly in women. The interval between gastric bypass and endoscopic procedure ranged from 1 to 144 months. There were no intraoperative complications. The rate of duodenal papillary cannulation was 100%. Regarding complications, the majority of cases were related to gastrostomy, and rarely to endoscopic procedure. There were two postoperative complications, a case of chest-abdominal pain refractory to high doses of morphine on the same day of the procedure, and a laboratory diagnosis of acute pancreatitis after the procedure in an asymptomatic patient. The maximum hospital stay was four days.

**Conclusion:**

The experience with endoscopic retrograde cholangiopancreatography through laparoscopic gastrostomy is a safe and effective procedure, since most complications are related to the it and did not altered the sequence to perform the conventional cholangiopancreatography.

## RESUMO

**Racional:**

A obesidade pode ser tratada com a cirurgia bariátrica; porém, doenças da via biliar como colelitíase e coledocolitíase podem surgir, principalmente devido à grande perda de peso. A colangiopancreatografia retrógrada é procedimento diagnóstico e terapêutico dessas afecções, e pode ser dificultada pela alteração anatômica após a operação.

**Objetivos:**

Relatar a eficácia e a segurança da colangiopancreatografia endoscópica retrógrada assistida por videolaparoscopia nos pacientes pós-cirurgia bariátrica com derivação gástrica em Y-de-Roux.

**Método:**

Estudo retrospectivo entre 2007 e 2017. Foram coletados: idade, gênero, indicação cirúrgica, tempo de internamento, diagnóstico etiológico, taxa de sucesso terapêutico, intercorrências intra e pós-operatórias.

**Resultados:**

Sete pacientes tiveram coledocolitíase confirmada por exame de imagem, principalmente em mulheres. O intervalo entre a derivação gástrica e o procedimento endoscópico variou de 1 a 144 meses. Não houve intercorrências intraoperatórias. A canulação da papila duodenal foi obtida em 100% dos casos. A maioria dos casos de complicações foi relacionada à gastrostomia, e raramente ao procedimento endoscópico. Houve duas intercorrências pós-operatórias, um caso de dor toracoabdominal refratária às doses elevadas de morfina no mesmo dia do procedimento, além de um diagnóstico laboratorial de pancreatite aguda pós-procedimento em paciente assintomático. A permanência hospitalar máxima foi de quatro dias.

**Conclusão:**

A colangiopancreatografia retrógrada endoscópica pela gastrostomia laparoscópica é método eficaz, seguro, visto que a maioria das complicações foi relacionada à gastrostomia; ela não altera a sequência de realização da colangiopancreatografia convencional.

## INTRODUCTION

**FIGURE 1:**
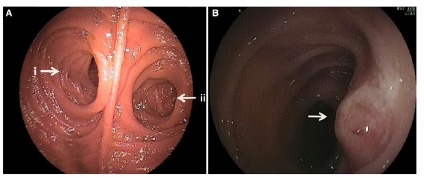
A) Appearance of the jejunal-jejunal anastomosis demonstrating the afferent (i) and efferent (ii) loops; B) endoscopy in the duodenum, with frontal view and oblique orientation of the papilla. Source: Amer (2015)^2^

The rapid weight loss due to bariatric surgery causes changes in bile composition, contributing to the formation of calculi^23^. Cholelithiasis develops in up to 38% of patients after six months of operation, and 41% of these patients become symptomatic^22^. Thus, endoscopic retrograde cholangiopancreatography (ERCP) may be indicated mainly for the treatment of stones in the bile duct after bariatric surgery. This procedure is made difficult by the greater length of the intestine up to the papilla (110-150 cm), by the different orientation of the papilla (enteroscope has the camera with frontal view, and the approach of the papilla becomes oblique - Figure 1 -, the conventional duodenoscope of CPRE has the lateral camera, and the approach of the papilla is frontal), adhesions and angulation of the intestine (>180 degrees in the enteroenteroanastomosis) or intestinal stenosis^2^ ^,^ ^22^ ^,^ ^23^. Thus, by the oral route, the ideal endoscope would be the one used in enteroscopy, but the accessories to perform ERCP in these cases have little availability due to the length and minor channel of instruments. To circumvent this situation, there are two ways to perform ERCP in bariatric patients with Y-de-Roux: those surgically assisted (open or laparoscopic surgery^16^) and by enteroscopy. The laparoscopic assisted ERCP hybrid technique (CAL) creates a videolaparoscopic gastrostomy that allows access of the duodenoscope to the excluded stomach. Efficacy in the literature is between 90-100% of the cases with a relatively low complication rate^2^ ^,^ ^13^ ^,^ ^14^ ^,^ ^26^. Some studies^7,21^ compared balloon enteroscopy-assisted ERCP (CAEB) with CAL. It had therapeutic superiority in bariatric patients with Roux-en-Y (59 vs. 100%, p<0.001, 56 vs. 100%, p<0.001) and no difference in complications (3.1 vs. 8.3% , p=0.392).

One of the largest studies is by Choi EK et al^7^, who analyzed 76 ERCPs by gastrostomy (CAL) and 32 ERCPs assisted by double balloon enteroscopy (CAEB) between 2005 and 2011. They showed a high therapeutic success rate (97%) in CAL groups, in addition to the superiority of the CAEB group (97% vs. 56%, p<0.001). The complication rate was higher in the CAL group (14.5% vs. 3.1%, p=0.022).

Thus, this paper aims to report the initial experience with CAL to treat lithiasis of biliary tract after gastric bypass in Roux-en-Y in bariatric patients.

## METHODS

The research project was approved by the Research Ethics Committee of the Pontifical Catholic University of Paraná, under protocol number 79295117.5.0000.0020. Patients submitted to this procedure were not approached for the preparation of this project, being exclusively the analysis of medical records, emphasizing the anonymity and confidentiality of the information collected.

The study site was in the Endoscopy Sector of Sugisawa Hospital Medical Center in Curitiba, PR, Brazil. The type was retrospective by means of the analysis of the electronic medical records of the hospital between January 2007 and January 2017. The following data were collected: age, gender, surgical indication, length of hospital stay, ERCP diagnosis, therapeutic success rate, intercurrences intra and postoperative.

The procedure protocol included prophylactic antibiotic therapy 1 h prior to abdominal access. Pneumoperitoneum was performed using a Veress needle in place without previous incisions, a blunt tip trocar and introduction of the laparoscopic camera. Inside the abdominal cavity, the bowel was followed to find Y-anastomosis, in addition to diagnosing other conditions such as internal hernias and adhesions. The biliopancreatic loop was clamped to prevent insufflation of the remainder of the gastrointestinal tract. Then the stomach or jejunum was circled with a pouch suture in the left upper quadrant of the abdomen. A 15- to 18-mm trocar was introduced into this quadrant, below the costal bed, where the duodenoscope was placed, usually in the great gastric curvature to facilitate the pylorus orientation. After the introduction, the endoscopist positioned himself on the left side of the patient. Endoscopy was continued through the stomach, either retrograde or via the jejunum, until the second duodenal portion and ERCP was performed. After removal of the endoscope, a second layer of suture was applied to prevent leakage of the gastrostomy. If the bowel had been incised, the suture should be transverse to reduce the risk of stenosis. The trocars were removed and skin/subcutaneous sutured and compressed by dressing. Therapeutic success was defined as performed sphincterectomy, extraction of the calculus and placement of pancreatobiliary stent.

### Statistical analysis

The data were stored in the Microsoft Word and Microsoft Excel programs for analysis. The results were presented in tables.

## RESULTS

The study included all patients diagnosed with choledocholithiasis after bariatric surgery with Roux-en-Y gastric bypass in 10 years back. Seven were identified through chart analysis. The mean age among them was 43.5 years, the majority being women. The time elapsed between gastric bypass and clinical presentation of choledocholithiasis was on average 6.5 years (Table 1).

**TABLE 1:** Data on patients undergoing ERCP (n=7)

Pacient	1	2	3	4	5	6	7
Gender	M	M	F	F	M	F	F
Age (years)	64	60	38	56	23	50	59
Δ Time Bypass ERCP (years)	1	10	2	10	2	6	12
Complications	No	No	No	Pain	Acute Pancreatites	No	No
Hospital Stay (days)	4	2	2	3	3	2	1

M = Masculino; F = Feminino.

The complaint of severe abdominal pain localized in the epigastrium or mesogastrium was reported by all patients. Two also had jaundice on admission. Three had already undergone cholecystectomy, one of whom had previously had a choledocholithiasis with a need for exploration of biliary tract.

The diagnosis of choledocholithiasis was confirmed by imaging: ultrasonography, computed tomography or abdominal magnetic resonance.

All patients underwent ERCP assisted by videolaparoscopy, according to the previously described technique with therapeutic success. The type of anesthesia employed was general. No intraoperative complications were reported.

Regarding postoperative complications, a case of thoracoabdominal pain that was refractory to high doses of morphine on the same day of the procedure was reported. Diagnostic hypotheses such as acute myocardial infarction and post-ERCP pancreatitis were excluded by examination. The pain had a good evolution, with complete remission on the same day. A laboratory diagnosis of acute pancreatitis after ERCP was described during the third postoperative day in an asymptomatic patient, remaining in clinical observation. Hospital stay was 1-4 days (mean 2.4 days) post-procedure (Table 1).

## DISCUSSION

In 1975 Schapira et al^20^ published the first case of ERCP via gastrostomy. The patient was a 67-year-old man with complicated mouth and tongue adenocarcinoma with proximal gastrointestinal stenosis due to radiotherapy. Thus, a gastrostomy tube for feeding was placed. He presented to the emergency department with abdominal pain and persistent jaundice. During the hospitalization, was removed the feeding tube, dilated the gastrostomy to 12 mm, allowing the passage of the duodenoscope through gastrostomy and performing ERCP.

In 1998 Baron and Vickers^4^ reported the first case of gastrostomy ERCP in patients with gastric bypass in Roux-en-Y due to recurrent pancreatitis. The advantages of this gastrostomy approach include a lower learning curve (it depends only on ERCP training) and can be performed regardless of the size of the handle made in bariatric surgery, as well as easier channeling and manometry.

Laparoscopic assisted ERCP has been a good alternative for the approach of patients after bariatric surgery with Roux-en-Y reconstruction. The main indications are the classic ones reported in the literature^3^ ^,^ ^6^ ^,^ ^7^ ^,^ ^8^ ^,^ ^14^ ^,^ ^17^ ^,^ ^19^. The seven patients who were treated by trans-gastric ERCP after Roux-en-Y gastric bypass had choledocholithiasis confirmed by some imaging examination.

The majority of the patients (57.1%) submitted to the procedure were women. This same pattern is observed in all articles in the literature. However, this data is not explored in the publications. It is suggested that rapid weight loss contributes to the formation of gallstones, but hormonal factors may contribute to its formation. Thus, cholelithiasis remains prevalent in females after bariatric surgery^24^.

 Although there are no technical difficulties or intraoperative complications in the CALs analyzed, some articles cite: the need for coordination between the endoscopist and the surgeon; sterilization of the endoscope and the transport of video tower and other materials to the operating room; lower endoscope response to lateral and in-out movements; orientation of the papilla slightly altered^19^.

The interval between gastric bypass and ERCP is variable, from months to years (1-242 months)^6^ ^,^ ^14^. In this study the time ranged from 1 to 144 months. The mean time of the procedure varies according to the experience of the service and/or intraoperative difficulties (41-245 min)^1^ ^,^ ^14^ ^,^ ^15^ ^,^ ^18^ ^,^ ^21^.

 Cannulation of the duodenal papilla is obtained in almost all cases (89-100%)^3^ ^,^ ^6^ ^,^ ^7^ ^,^ ^8^ ^,^ ^14^ ^,^ ^17^ ^,^ ^21^, which was also found in the present study, with 100% success of the technique. In addition, the gastrostomy route is superior to the technique of double balloon enteroscopy^7^ ^,^ ^12^, since it has no lateral vision, making it difficult to canalize the papilla and doing therapeutic procedures. In addition, specific equipment is required for the enteroscope, a longer operative time - which is reasonable for treating gallstone disease of the bile ducts -, but unsuitable for pancreatic diseases or dysfunction of the sphincter of Oddi. The only advantage of enteroscopy is to perform it with ERCP in only one time.

 There are several therapeutic possibilities that can be performed after adequate visualization of the papilla^3^ ^,^ ^6^ ^,^ ^7^ ^,^ ^8^ ^,^ ^14^ ^,^ ^17^ ^,^ ^21^. In addition, because videolaparoscopy is realized, it is possible to diagnose and correct internal hernias, and also to perform cholecystectomy in the same procedure^16^.

Regarding complications, most of them are related to gastrostomy and rarely to ERCP (pancreatitis, bleeding, perforations, stent migration, cholangitis)^1^ ^,^ ^3^ ^,^ ^7^ ^,^ ^8^ ^,^ ^9^ ^,^ ^14^ ^,^ ^17^. Regarding the case of thoracoabdominal pain refractory to high doses of morphine on the same day of the procedure, no similar description was found in literature.

The length of hospital stay usually does not exceed three days if the procedure is free of complications. However, hospitalization can be prolonged - up to 22 days - in complications^1^ ^,^ ^5^ ^,^ ^7^ ^,^ ^8^ ^,^ ^9^ ^,^ ^10^ ^,^ ^14^ ^,^ ^15^ ^,^ ^17^ ^,^ ^21^ ^,^ ^25^. The maximum hospital stay described in this study was four days, being in consonance with other published studies.

## CONCLUSION

Endoscopic retrograde cholangiopancreatography by laparoscopic gastrostomy is an effective, safe method, since most of the complications are related to gastrostomy; it does not alter the sequence of performing conventional cholangiopancreatography.
